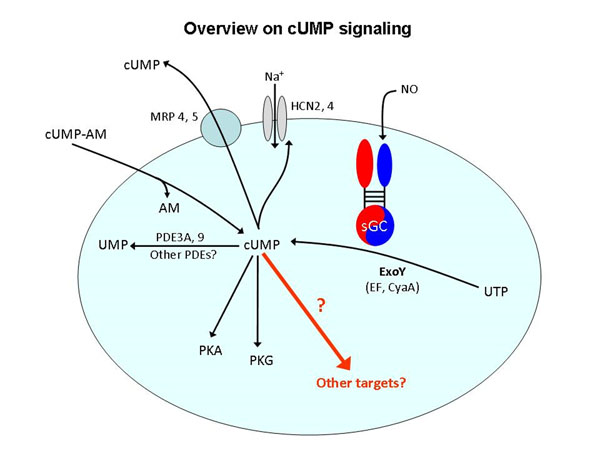# *Pseudomonas aeruginosa* ExoY, a cyclic GMP- and cyclic UMP-generating nucleotidyl cyclase

**DOI:** 10.1186/2050-6511-14-S1-O36

**Published:** 2013-08-29

**Authors:** Roland Seifert, Christina Hartwig, Sabine Wolter, Daniel Reinecke, Heike Burhenne, Volkhard Kaever, Antje Munder, Burkhard Tümmler, Frank Schwede, Manuel Grundmann, Evi Kostenis, Dara W Frank, Ulrike Beckert

**Affiliations:** 1 Institute of Pharmacology, Hannover Medical School (MHH), Germany; 2 Core Unit Mass Spectrometry & Metabolomics, MHH, Germany; 3 Department of Pediatric Pneumology, Allergology and Neonatology, MHH, Germany; 4 Biolog Life Science Institute, Bremen, Germany; 5 Institute of Pharmaceutical Biology, University of Bonn, Germany; 6 Department of Microbiology & Molecular Genetics, Medical College of Wisconsin, Milwaukee, USA

## Background

In previous studies we showed that in addition to the cyclic nucleotides (cNMPs) cAMP and cGMP, mammalian cells also contain cCMP and cUMP [for review see [1], [2]]. cCMP and cUMP are generated by the bacterial exotoxins CyaA from *Bordetella pertussis* and edema factor (EF) from *Bacillus anthracis* as well as nitric oxide (NO)-stimulated soluble guanylyl cyclase (sGC). cCMP and cUMP activate cAMP-dependent protein kinase (PKA), cGMP-dependent protein kinase (PKG) and cyclic nucleotide-regulated ion channels (HCN channels). cUMP, but not cCMP, is degraded by several known phosphodiesterases (PDEs). cCMP and cUMP are also exported from cells via multidrug resistance proteins (MRPs 4 and 5). These findings suggest that cCMP and cUMP constitute second messenger molecules with distinct signalling properties.

*Pseudomonas aeruginosa* is an important pathogenic bacterium, specifically in patients with cystic fibrosis. *P. aeruginosa* injects effector proteins into host cells via the type III secretion system and, thereby, manipulates their functions. One of the effector proteins of *P. aeruginosa* is ExoY. ExoY possesses structural similarity with CyaA and EF in the catalytic domain and was assumed to constitute an adenylyl cyclase (AC) [3]. However, the cAMP-forming capacity of ExoY is rather low. More recently, it has been shown that ExoY is capable of producing large quantities of cGMP [4]. On this background, we examined the question whether ExoY also exhibits cytidylyl- and uridylyl cyclase activity.

## Methods

We transfected mammalian cells with a plasmid encoding ExoY or a catalytically inactive ExoY mutant (ExoY-M). We also infected cells with *P. aeruginosa* encoding ExoY or ExoY-M. In addition, we infected mice intratracheally with *P. aeruginosa*. We determined cNMP concentrations with highly sensitive and specific HPLC-MS/MS and HPLC-MS/TOF methods. We assessed cell morphology, apoptosis and label-free dynamic mass distribution, constituting a holistic and unbiased assay for cell activation.

## Results

In all systems studied, we observed striking increases in cUMP concentrations by ExoY but not by ExoY-M. The cUMP increase was particularly large in lung tissue following infection. One of the reasons for the huge cUMP increase is the fact that in mouse lung, the activity of cUMP-degrading PDE is only low. Increases in cCMP and cGMP were less pronounced. ExoY induced morphological changes and cell death. Biological effects of ExoY were mimicked by the cell membrane-permeable cUMP analog, cUMP-acetoxymethylester. The biological effects of cUMP can only be partially explained by activation of PKA and PKG, indicating that additional and as yet unknown target proteins are involved in cUMP signal transduction. This was particularly evident in the dynamic mass distribution experiments.

## Conclusion

In contrast to previous assumptions, ExoY is not an AC but rather a nucleotidyl cyclase with strong preference for cGMP- and cUMP production. The large cUMP increases in the lung following infection with *P. aeruginosa* suggest that this cNMP plays a specific role in signal transduction and in the pathophysiology of lung function. Therefore, current research efforts are directed towards the identification of specific cUMP-binding proteins using the cNMP agarose affinity technique [5]. Since there are striking differences in degradation of cUMP and cGMP in the lung, we assume that the two cNMPs exhibit different functions. Based on the successful uses of PDE5 inhibitors and sGC activators and stimulators in the therapy of lung diseases, we envisage substantial clinical potential for cUMP-modulating drugs.

**Figure 1:**